# A dielectrophoresis-based microfluidic system having double-sided optimized 3D electrodes for label-free cancer cell separation with preserving cell viability

**DOI:** 10.1038/s41598-022-16286-0

**Published:** 2022-07-15

**Authors:** V. Varmazyari, H. Habibiyan, H. Ghafoorifard, M. Ebrahimi, S. Ghafouri-Fard

**Affiliations:** 1 Electrical Engineering Department, Amirkabir University of Technology, Tehran, Iran; 2 Energy Engineering and Physics Department, Amirkabir University of Technology, Tehran, Iran; 3 Department of Stem Cells and Developmental Biology, Cell Sciences Research Center, Royan Institute for Stem Cell Biology and Technology, ACECR, Tehran, Iran; 4 Department of Medical Genetics, Shahid Beheshti University of Medical Sciences, Tehran, Iran

**Keywords:** Biomedical engineering, Lab-on-a-chip

## Abstract

Early detection of circulating tumor cells (CTCs) in a patient's blood is essential to accurate prognosis and effective cancer treatment monitoring. The methods used to detect and separate CTCs should have a high recovery rate and ensure cells viability for post-processing operations, such as cell culture and genetic analysis. In this paper, a novel dielectrophoresis (DEP)-based microfluidic system is presented for separating MDA-MB-231 cancer cells from various subtypes of WBCs with the practical cell viability approach. Three configurations for the sidewall electrodes are investigated to evaluate the separation performance. The simulation results based on the finite-element method show that semi-circular electrodes have the best performance with a recovery rate of nearly 95% under the same operational and geometric conditions. In this configuration, the maximum applied electric field (1.11 × 10^5^ V/m) to separate MDA-MB-231 is lower than the threshold value for cell electroporation. Also, the Joule heating study in this configuration shows that the cells are not damaged in the fluid temperature gradient (equal to 1 K). We hope that such a complete and step-by-step design is suitable to achieve DEP-based applicable cell separation biochips.

## Introduction

Metastasis is generally considered the most important clinical indicator of cancer. Over ninety percent of all cancer-related deaths are directly attributed to metastasis^1^. The dispersal of tumor cells through the circulatory system is a critical step in the metastatic process. These circulating tumor cells (CTCs) may release at the early stages of the disease, which is the basis for the later development of metastasis^2^. Detection and enumeration of CTCs is a promising field for assessing metastatic progress, and tracking the performance of cancer treatment^3^. Due to the very low concentration of CTCs (only several cells per mL) against the high background of blood cells (approximately 5 × 10^9^ RBCs per mL and 5 × 10^6^ WBCs per mL), we need to separate them from a blood sample with high sensitivity on a single-cell level and acceptable throughput^4–7^.

Fluorescence-activated cell sorting and magnetic-activated cell sorting are conventional methods for separating CTCs from blood samples based on surface markers. However, there is considerable cell loss (~ 20–40%) due to the inability of these methods to detect CTCs with a reduced EpCAM expression (e.g. CTCs with epithelial to mesenchymal transition (EMT) or cancer stem cell (CSC))^8^. Label-free-based techniques separate a variety of CTCs by removing surface marker bias. Lab-on-a-chip (LOC) platforms are exploited for a wide range of applications due to their fundamental attributes. Microfluidic applications implemented in LOC devices have been rapidly expanded within the last two decades.

A variety of technologies based on microfluidic systems have been proposed to manipulate and separate cells in an aqueous solution, including mechanical, inertial, hydrodynamic, acoustic, optical, magnetic, and electrical methodologies^9^. Among these approaches, dielectrophoresis (DEP) technology has gained significant attention for separating cancer cells. Unlike other techniques, DEP devices depend on the dielectric properties, representing the structural, morphological, and chemical characteristics of bioparticles and allowing highly selective and sensitive analysis^10^. DEP-based manipulation can be fully controlled by varying the electrical conductivity and permittivity of the suspending medium or the frequency and intensity of the applied electric field. In this method, alternating field (AC) is used to minimize electrolysis reactions of microelectrodes and Joule heating problems^11^. DEP-based systems are easily integrated with LOC devices due to their effective interface with conventional electronics.

Efficient separation of CTCs and cells viability are crucial for post-processing analysis. It is noteworthy that the spatially non-uniform electric field of the microelectrodes and the DEP force are used to concentrate the target cells in the desired regions. The intensity of the electric field is often high near the electrodes. When a cell enters a region with a high electric field, the cell membrane is temporarily disrupted and the intracellular components including DNA, RNA, proteins, and organelles are released. This phenomenon is called electroporation. Although electroporation is widely used in applications such as drug delivery, cancer treatment, and gene therapy, if the cell is lysed during the separation unit, the intracellular components may come out of the unwanted outlet. Therefore, in the DEP-based separation, it is necessary to minimize the applied electric field throughout the microchannel for efficient separation without cell lysis. The configuration of the electrodes plays an important role in the electric field applied to the cells.

The preservation of cell viability after the separation step is an important issue that has not been completely addressed in many previous reports. In this paper, a DEP-based microfluidic device is proposed to separate MDA-MB-231 cancer cells from various subtypes of WBCs. The main purpose is to prevent damage to target cells during the separation process. In the first stage, three designs, triangular, rectangular, and semi-circular, are considered for the 3D sidewall electrodes configuration due to the critical role of electrodes configuration in determining the electric field applied to the cells. Under similar operational and geometric parameters, the electric field applied to the cells inside the microchannel is investigated. The semi-circular design is the final structure among the mentioned configurations to achieve cell viability and prevent cell electroporation. In the next step, the temperature distribution within the microchannel and its effect on cell viability in the semi-circular configuration is evaluated. To the best of our knowledge, such a study on the configuration of electrodes with a cell viability approach for post-processing analysis has not been previously reported.

It should be noted that our proposed design has several innovations such as (a) The novelty of the proposed system's geometrical structure, (b) Considering the maximum electric field that can be applied to cells during the separation process of two different cells (CTCs from WBCs) without destruction or electroporation, (c) Design and optimization of the configuration of the 3D sidewall electrodes adjacent to the channel wall, and (d) Investigation of Joule heating and its effect on electrode design. These points are not entirely and step by step presented in similar papers. Our paper will help researchers consider these issues in their system design before fabrication and carrying out biological tests.

## Materials and methods

### Proposed system

**Figure 1:**
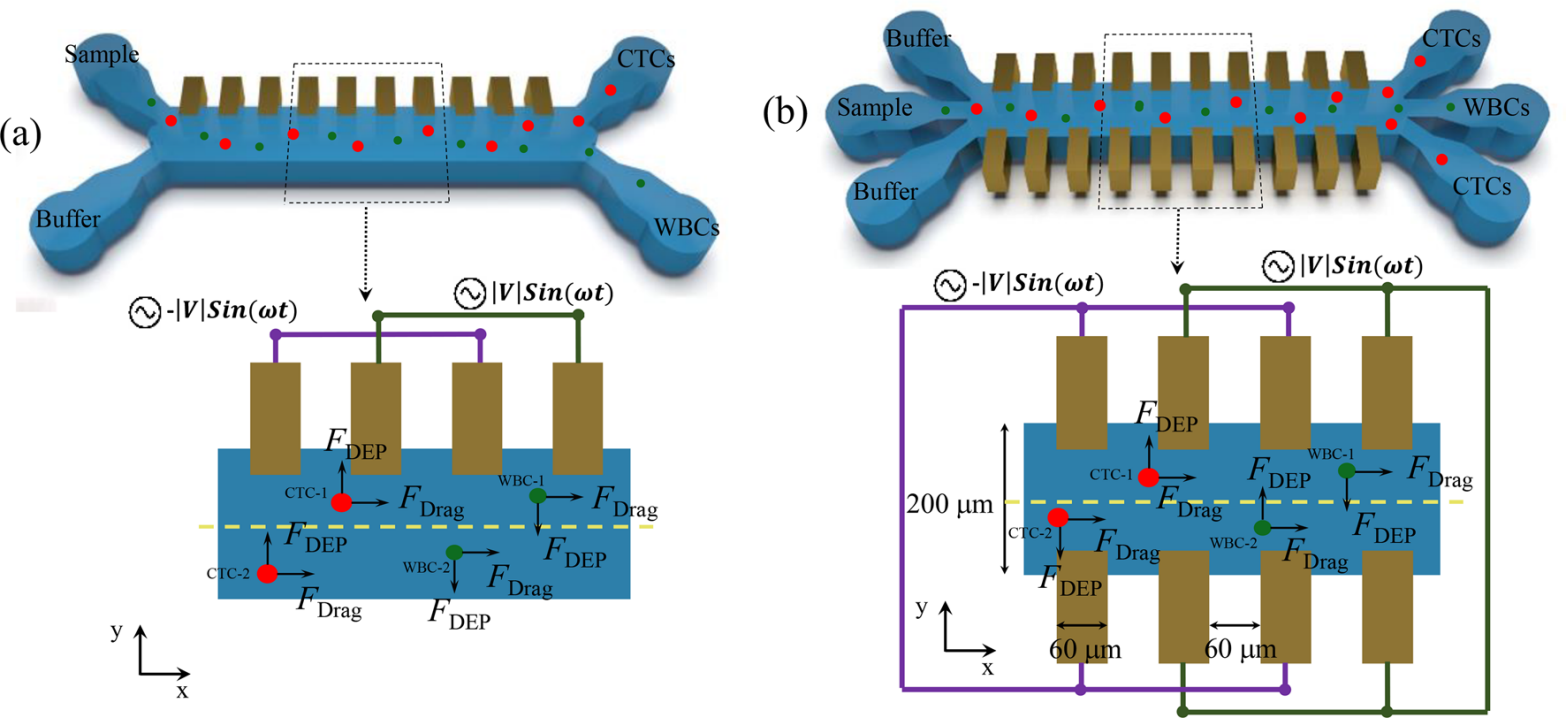
Schematic view of the (**a**) conventional DEP-based systems (**b**) proposed cell separation system. Electrodes are excited by sinusoidal voltages with a 180° phase difference to apply DEP force on cells.

Figure 1 illustrates the structure of the proposed PDMS-based microfluidic device with 3D sidewall electrodes compared with a conventional platform. The microchannel system consists of the main channel with a width (W) of 200 μm, a height (H) of 200 μm, and a length (L) of 1.35 mm, three inlets (sample and buffer solution inlets), and three outlets. The sample inlet is for injecting cells, while buffer inlets force the cells entering from the sample inlet to the middle of the microchannel. The outlet arms were designed after studying the flow behavior of the cells so that the WBCs are collected in the middle outlet and the cancer cells in the two side outlets. The electrodes are arranged on the microchannel's sidewalls to create a non-uniform distribution of the electric field (to improve cell separation sensitivity) along the channel. Compared to the planar electrode array at the bottom of the channel, the great advantage of this method is coverage of the entire channel space by the applied electric field. Various materials have been proposed for implementing sidewall electrodes in microfluidic devices, including metal^12^, carbon^13^, polymer^14^, and silicon^15^. In our simulations, sidewall metal electrodes (Au) have been used to apply voltage to the microchannel. Electrodes’ width and their gaps are 60 μm. The supplementary materials contain details on the fabrication process.

In our proposed system, 3D electrodes are placed on both sides of the microchannel, so the device has three inlets (two inlets for buffer and one inlet for sample) and three outlets (two outlets for CTCs and one outlet for WBCs). Whereas in conventional DEP-based systems, these electrodes are located on one side of the microchannel and the system has two inlets (one inlet for the buffer and one inlet for the sample) and two outlets (one outlet for the CTCs and one outlet for the WBCs). Figure 1 shows the location of several CTCs and WBCs at different positions inside the microchannel to understand the difference between the operation of conventional devices and the suggested system. As shown in Fig. 1a, if the distance between the target cells and the electrodes is more than half of the microchannel width (CTC-2), it is necessary to increase the applied DEP force to return them to the desired path (corresponding to the increase in voltage applied to the electrodes), as a result, the target cells near the electrodes (CTC-1) will be exposed to high electric fields, and the probability of damage will increase. To overcome the extra applied electric field, the channel width can be designed smaller, which leads to a lower recovery rate due to the exit of target cells from the unwanted outlet. The cells exit from undesirable output will be even greater if they collide near the outlets and are transferred to the unwanted path. For example, if a WBC is located near the outlets at the place of WBC-1 (e.g. due to the collision between cells), there may not be enough time for the DEP force to act on the cell to transfer it to the desired outlet. The proposed system (Fig. 1 b) does not have these problems due to 3D electrodes on both sides of the microchannel and three inputs and outputs. For example, if a WBC is in the place of WBC-1 or WBC-2 for any reason, it is pushed to the center of the channel and the desired outlet by the DEP force. Also, if a CTC is in the place of CTC-1 or CTC-2, it doesn’t need to move to the opposite half of the channel width. This CTC is going out from the desired outlet on the sides of the channel by the DEP forces shown in Fig. 1b. According to these explanations, it can be said that the performance of the proposed system is the same as two conventional parallel designs, and as a result, the throughput of the present design is much better than conventional designs.

**Figure 2:**
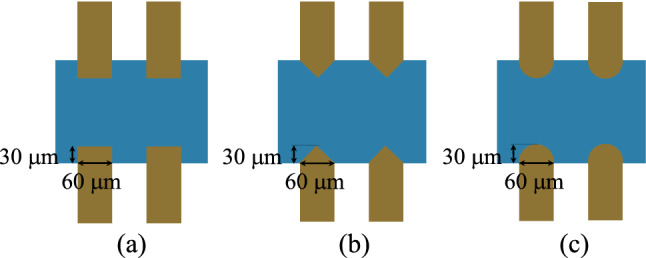
The configuration of investigated electrodes: (**a**) rectangular (**b**) triangular (**c**) semi-circular.

DEP mechanism separates cells with different diameters based on dielectric properties. The DEP-based system should be designed so that the cells are not damaged, an important issue that has not been addressed in many suggested devices^16,17^. Since the DEP force applied to a cell is proportional to its cube radius, the amount of voltage applied to the electrodes must be selected so that the entering CTCs lead to the desired output. Due to the random placement of CTCs at the microchannel inlet, it is possible for the CTCs to move to the channel's sides. As will be mentioned later, at the sides of the microchannel and near the electrodes, the CTCs experience a larger electric field and may be damaged. Also, the configuration of electrodes has a significant effect on electric field distribution near the microchannel walls. In most DEP-based studies, rectangular electrodes have been used to apply DEP force. In our proposed system, three configurations of electrodes including rectangular, triangular, and semi-circular with a CTCs viability approach are investigated. Figure 2 shows these configurations along with the dimensions of the electrodes. The dimensions of the electrodes are considered similar to compare the effect of the generated electric field.

By interacting biological cells with electric fields in microfluidic systems, they potentially experience some electrokinetic mechanisms. The two electrical mechanisms that act directly on cells are dielectrophoresis and electroporation. These two mechanisms are covered in the following subsections.

### Dielectrophoresis

Dielectrophoresis refers to the motion of the polarized cell in a dielectric medium under a spatially non-uniform electric field. The morphology and frequency of the applied electric field determine the degree of polarization of the cells. The DEP force applied to cells depends on the physical properties of the cells and the medium, such as permittivity and conductivity. The general expression for the DEP force exerting on a cell is expressed as

1 $$F_{DEP} = 2\pi \varepsilon_{m} r^{3} Re\left\{ {F_{CM} } \right\}\nabla E_{rms}^{2}$$

here $\varepsilon_{m}$ is the absolute medium permittivity, *r* is the cell radius, $E_{rms}$ is the root mean square value of the electric field and $Re\left\{ {F_{CM} } \right\}$ is the real part of Clausius–Mossotti (CM) factor given by:

2 $$F_{CM} = \frac{{\varepsilon_{cell}^{*} - \varepsilon_{m}^{*} }}{{\varepsilon_{cell}^{*} + 2\varepsilon_{m}^{*} }}$$

where $\varepsilon_{cell}^{*}$ and $\varepsilon_{m}^{*}$ are complex permittivity of the cell and medium, respectively, and is given by,

3 $$\varepsilon^{*} = \varepsilon - j\frac{\sigma }{\omega }$$

where $\varepsilon$ is the permittivity, $\sigma$ is the conductivity, $j$ is the imaginary unit, and $\omega$ is the angular frequency. The $F_{CM}$ determines the sign of the DEP force and the direction of the cell's motion. Cells with different dielectric properties experience various DEP forces depending on the frequency applied to the electrodes. The real part of $F_{CM}$ is in the range of − 0.5 to 1. Positive $Re\left\{ {F_{CM} } \right\}$ leads to a positive DEP force (pDEP) on the cells and pushes them towards the high electric field zones. In this case, the cell exhibits more polarizability than the medium. On the other hand, a negative value for $Re\left\{ {F_{CM} } \right\}$ causes a negative DEP force (nDEP) and drives the cells away from the high electric gradient. At specific frequencies, called “*crossover frequencies (f*_*xo*_*)*,” the value of $Re\left\{ {F_{CM} } \right\}$ is zero, and, as a result, DEP force is zero.

As shown in Fig. 1, the effective DEP force is the $F_{{DEP_{Y} }}$ in separating cells according to the proposed structure. The magnitude of DEP force acting on the cell changes in the y-direction as follows:

4 $$F_{{DEP_{Y} }} = 2\pi \varepsilon_{m} r^{3} Re\left\{ {F_{CM} } \right\}\nabla E_{y}^{2}$$

### Electroporation

Exposing a cell to an electric field induces a voltage in its membrane (induced transmembrane voltage, $V_{c}$). Applying a sufficiently strong electric field to a cell can lead to transmembrane voltage far exceeding the cell physiological range. As a result, non-physiological effects may occur, including structural rearrangements of lipids in the membrane bilayer resulting in the formation and stabilization of pores, which alter the dielectric properties of the cell. This phenomenon is termed *electroporation*. Analytical description of the steady-state of transmembrane voltage induced on cells by sinusoidal electric fields is expressed as follows^18,19^:

5 $$V_{c} = f_{c} ERcos\theta \frac{{1 + j\omega \tau_{m2} }}{{1 + j\omega \tau_{m1} }}$$

6 $$f_{c} = \frac{{3\sigma_{e} \left[ {3\delta R^{2} \sigma_{cyt} + \left( {3\delta^{2} R - \delta^{3} } \right)\left( {\sigma_{mem} - \sigma_{cyt} } \right)} \right]}}{{2\left[ {R^{3} \left( {\sigma_{mem} + 2\sigma_{e} } \right)\left( {\sigma_{mem} + \frac{1}{2}\sigma_{cyt} } \right) - \left( {R - \delta} \right)^{3} \left( {\sigma_{e} - \sigma_{mem} } \right)\left( {\sigma_{cyt} - \sigma_{mem} } \right)} \right]}}$$

7 $$\tau_{m1} = \frac{{\varepsilon_{mem} }}{{\frac{\delta}{R}\times\frac{{2\sigma_{cyt} \sigma_{e} }}{{\sigma_{cyt} + 2\sigma_{e} }} + \sigma_{mem} }}$$

8 $$\tau_{m2} = \frac{{\varepsilon_{cyt} + 2\varepsilon_{e} }}{{\sigma_{cyt} + 2\sigma_{e} }}$$

where $R$ is the cell radius, $E$ is the electric field intensity, $\tau_{m1}$ and $\tau_{m2}$ are the membrane's first and second time constant for describing the frequency dependence of the $V_{c}$, and $\theta$ is the angle of the given membrane position and the electric field direction. Also $\sigma_{cyt}$, $\sigma_{mem}$ and $\sigma_{e}$ are the conductivities of the cytoplasm, the cell membrane, and the external medium, respectively, and $\delta$ is the membrane thickness.

Electroporation can be reversible or irreversible depending on the degree of the membrane's structural changes. The pores heal and reseal the bilayer when the electric field is removed in the reversible case. In contrast, in the irreversible case, the pores expand too much, leading to mechanical rupture of the cell membrane^20^.

In some papers, the cells are trapped at a location of the microchannel using DEP force. Then, the trapped cells are electroporated by applying a high electric field^21,22^.

## Results and discussion

### Numerical model description

To evaluate the performance of the proposed system, the finite element method (FEM) and COMSOL Multiphysics (COMSOL Inc., Burlington, MA, USA) are used to model the flow field, the electric field, particle tracing, and Joule heating within the microchannel. The proposed structure needs to be discretized for finite element analysis based on triangle meshes of different sizes. Triangle shapes are used to approximate the structural displacement field. For around areas of the electrodes, finer meshes are assigned due to the expected higher electrical field gradients. Therefore, meshing with physics-controlled elements was chosen. For mesh independency studies, the velocity profile in the microchannel was considered to select the optimum mesh size that saves computation time in producing the estimated outcome. To this end, four different mesh sizes of fine, normal, coarse, and coarser were considered. Simulation results with these mesh sizes showed that the velocity profiles obtained from fine and normal meshes have less than a 5% difference. Thus, the normal mesh is used for the computational results presented in this paper. The meshing evaluation performed on the proposed structure and the velocity profile for different meshes are shown in Figs. S2 and S3 of the Supplementary Information. It should be noted that all simulations performed in the proposed system were done in 3D mode. However, 2D simulations were tested for the trajectory of cells, which did not differ significantly from the 3D results. Three steps are performed to investigate cell trajectories: Initially, the continuum fluid dynamics is evaluated by solving the linear incompressible Navier–Stokes equation. The associated boundary conditions are: (1) the channel wall is a non-slip boundary, (2) the inlet fluid is controlled by the flow rate, (3) the fluid at the outlet is controlled by the pressure, and the pressure at the outlet is set to be atmospheric pressure, that is P = 0. Following that, the electric field distribution for the given geometry is computed using the Laplace equation. The corresponding boundary conditions are: (1) the wall surface, inlet, and outlet of the microchannel are insulated boundary conditions, and (2) sinusoidal AC voltages with a 180° phase difference are exploited to the array of electrodes. Finally, the cell trajectories in the microfluidic system are calculated using Newton's second law under the combination of the different applied forces and fluid flow. The related boundary conditions include: (1) the channel wall is a rebound type, and (2) the initial velocity of the inlet fluid is based on the velocity field in the medium. According to Newton’s second law, the balance of many forces determines the trajectory of a cell in a DEP-based microfluidic system with a laminar flow, which is described as follows^23,24^:

9 $$\frac{d}{dt}\left( {m_{c} {\mathbf{v}}} \right) = {\mathbf{F}}_{{\mathbf{t}}}$$

10 $${\text{F}}_{{\text{t}}} = {\text{F}}_{{{\text{DEP}}}} + {\text{F}}_{{{\text{Drag}}}} + {\text{F}}_{{{\text{Virtual}}\;{\text{Mass}}}} + {\text{F}}_{{{\text{Lift}}}} + {\text{F}}_{{{\text{Gravity}}}} + {\text{F}}_{{{\text{Brownian}}}} + {\text{F}}_{{{\text{Basset}}}}$$

Many of these forces could be ignored for cell separation applications, depending on the size of the cell and the intensity of the electric field. In a relatively weak electric field, drag force is dominant, while gravitational and dielectrophoresis forces have comparable order of magnitude. But in a relatively high electric field, drag force and dielectrophoresis force are the most dominant forces, and other forces could be ignored^22^. Most of the studies reported in the literature only consider the two most significant forces: the drag force and the dielectrophoresis force^25^. In this study, in addition to DEP and Drag forces, lift and gravity forces are also considered to determine the cells trajectory. More details are provided in Supplementary Information.

To validate the FEM solver, the study of Zhang et al. was considered in which the electric field distribution, the spectrogram of the CM factor, and the cell trajectories under different applied voltages were regenerated^26^. There was a good agreement between our simulation results and the results reported in mentioned reference. After ensuring the accuracy of the simulation method, the proposed DEP-based devices were modeled in a completely similar manner. Also, an attempt was made to express the study's innovation to maintain cell viability for post-processing analysis and resolving problems associated with conventional systems.

### Cell dielectric modeling

**Figure 3:**
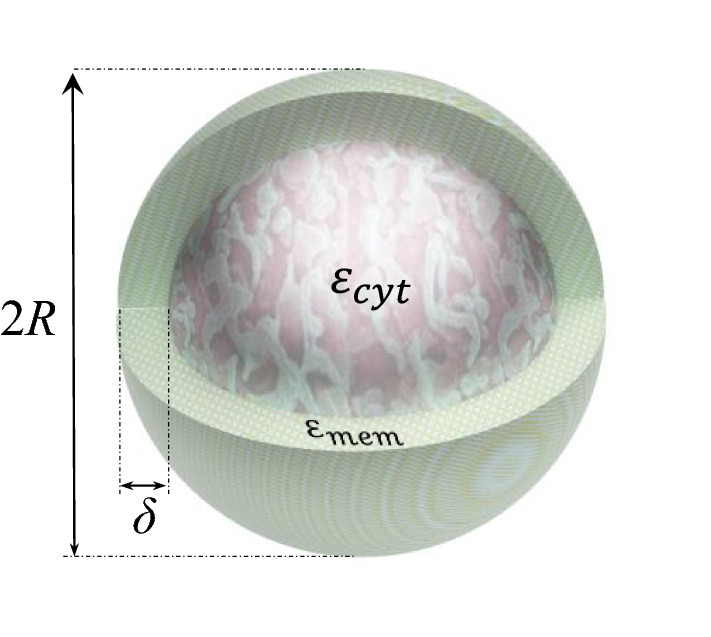
Single shell model of a cell.

The main challenge of cancer cells’ analysis is to separate CTCs from WBCs because size-based methods can easily separate other blood parameters due to their smaller size. In this study the considered cell suspension is composed of MDA-MB-231 cells as a breast cancer cell and the different subtypes of WBCs. MDA-MB-231 are triple-negative breast cancer cells, which are difficult to treat. This type of cancer is more common in young women as well as African American women^27^. The common approach to evaluate the electrical characteristics of biological cells is using a single-shell model. As illustrated in Fig. 3, the cell behaves as a homogeneous spherical particle in which the cytoplasm is surrounded by a thin layer of lipid membrane^28^. In this model, the effective complex permittivity of the cell can be calculated by following relation^29^:

11 $$\varepsilon_{cell}^{*} = \varepsilon_{mem}^{*} \left\{ {\frac{{\left[ {\left( {R + \delta } \right)/R} \right]^{3} + 2\left[ {\left( {\varepsilon_{cyt}^{*} - \varepsilon_{mem}^{*} } \right)/\left( {\varepsilon_{cyt}^{*} + 2\varepsilon_{mem}^{*} } \right)} \right]}}{{\left[ {\left( {R + \delta } \right)/R} \right]^{3} - \left[ {\left( {\varepsilon_{cyt}^{*} - \varepsilon_{mem}^{*} } \right)/\left( {\varepsilon_{cyt}^{*} + 2\varepsilon_{mem}^{*} } \right)} \right]}}} \right\}$$

where $R$ is the cell radius, $\delta$ is the cell membrane thickness, $\varepsilon_{cyt}^{*}$ and $\varepsilon_{mem}^{*}$ are the complex permittivites of the cytoplasm and the membrane, respectively. The conductivity and relative permittivity of the suspension medium are 55 mS/m and 80, respectively.

As shown in Fig. 1, the buffer is injected via two inlets of the DEP system and contain a large portion of the fluid inside the microchannel. Therefore, the suspension used in the simulations is assumed as a Newtonian fluid. This solution can be prepared by adding Sucrose (C_12_H_22_O_11_) and Dextrose (C_6_H_12_O_6_) to distilled water in the ratio of 8.5% w/w and 0.3% w/w, respectively^30,31^, or can be prepared by phosphate buffer saline (PBS) diluted in sucrose solution^32^. Also, the cells were considered as spherical particles with the same size and electrical properties found in the previous literature. Dielectric and mechanical properties of different subtypes of WBCs and MDA-MB-231 are summarized in Table 1.

**Table 1:** Dielectric and mechanical properties of selected cells.

Cell type	$R$(µm)	$\varepsilon_{mem}$	$\sigma_{mem } \left( {\frac{{\text{s}}}{{\text{m}}}} \right)$	$\varepsilon_{cyt}$	$\sigma_{cyt } \left( {\frac{{\text{s}}}{{\text{m}}}} \right)$	$\delta \left( {{\text{nm}}} \right)$	References
Granulocytes	4.71 ± 0.23	5	1 × 10^–6^	151	0.6	4	^33^ ^34^ ^35^
T-lymphocytes	3.29 ± 0.35	5	1 × 10^–6^	104	0.65	4	
B-lymphocytes	3.29 ± 0.26	5	1 × 10^–6^	154	0.73	4	
Monocytes	4.63 ± 0.36	5	1 × 10^–6^	127	0.56	4	
MDA-MB-231	6.2 ± 0.58	11.75	1 × 10^–6^	52	0.62	4	

### Cell separation using DEP system

This paper investigates the performance of a proposed DEP device for three different configurations of 3D electrodes to separate MDA-MB-231 cancer cells from the different subtypes of WBCs with a cell viability approach. It should be noted that WBCs and CTCs are slightly larger than other blood components, so before separating WBCs from CTCs in the proposed system, they can be separated from other blood components using these two methods: (1) conventional cell sorting techniques such as centrifugation with density gradient, and (2) cell sorting based on microfluidic systems using deterministic lateral displacement (DLD)^36^, pinch flow fractionation (PFF)^37^, hydrodynamic filtration^38^, and inertial migration^39^ mechanisms. In this section, various parameters such as cell trajectory, the electric field in the microchannel, DEP force applied to cells, and Joule heating are surveyed.

**Figure 4:**
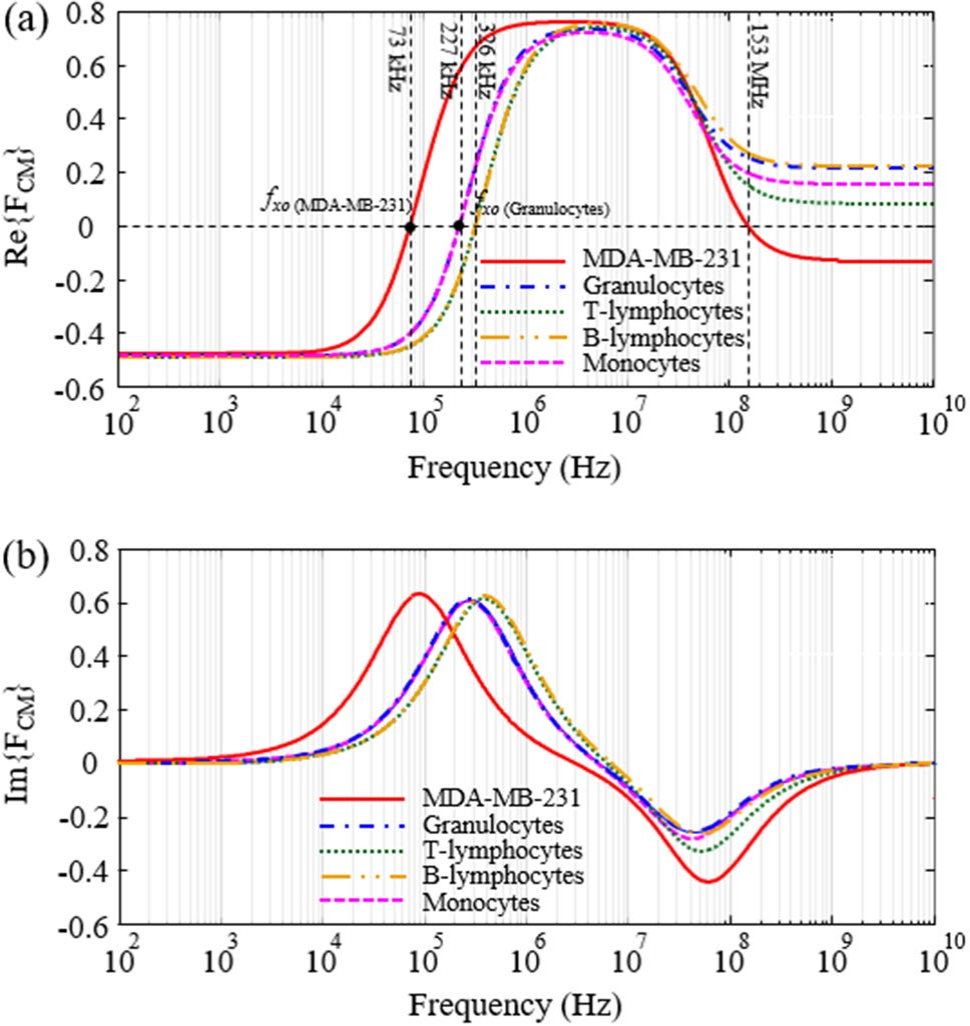
(**a**) Real part and (**b**) Imaginary part of CM factor as a function of applied electric field frequency for MDA-MB-231 and different subtypes of WBCs.

First, it is necessary to determine the frequency of the voltage source applied to the electrodes for efficient separation of CTCs. Figure 4 shows the real and imaginary parts of the CM factor as a function of applied electric field frequency for MDA-MB-231 and different subtypes of WBCs. These graphs are plotted based on Table 1 and equations of (2) and (11). As can be seen, the crossover frequency of MDA-MB-231 is 73 kHz and the smallest crossover frequency in the different subtypes of WBCs is the Granulocytes with a value of 227 kHz. The sign of the DEP force alters at the crossover frequency. Therefore, the electric field frequency of 125 kHz is considered to apply positive and negative DEP forces to MDA-MB-231 and WBCs, respectively. Due to the fact that the crossover frequency of RBCs and platelets is higher than WBCs, even if these cells enter the microchannel, the performance of the proposed system will not be disrupted^26^. Because, like WBCs, at the mentioned frequency (125 kHz), a negative DEP force is applied to these cells results to exit the WBCs outlet.

**Figure 5:**
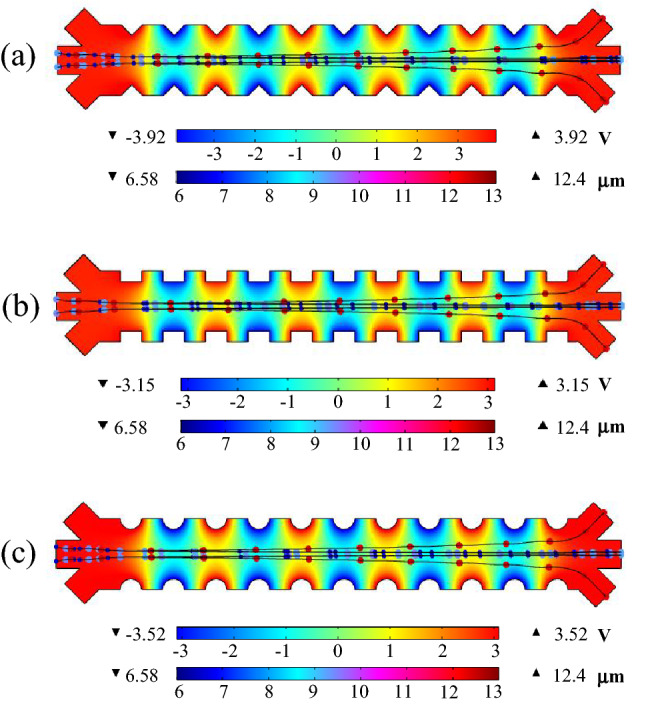
Spatial variation of the electric potential and trajectories of MDA-MB-231 and WBCs by applying the dielectrophoresis force for various electrode configurations (**a**) triangular (**b**) rectangular (**c**) semi-circular. The top color bar shows the applied voltage to the sidewall electrodes and the bottom color bar indicates the diameter of the cells.

The cells' behavior is investigated in the proposed channel by fixing the applied frequency to the electrodes (125 kHz). To this end, a mixed sample of MDA-MB-231, Granulocytes, T-lymphocytes, B-lymphocytes, and Monocytes is delivered into the suggested device, and their trajectories are captured in the presence of an applied electric field. Numerous simulations have been performed for the three mentioned electrode configurations to evaluate the proper performance of the system. In each case, different voltages have been applied to the electrodes and the minimum voltage has been selected for the effective separation of CTCs. Spatial variation of the electric potential and trajectories of MDA-MB-231 and WBCs by applying the dielectrophoresis force for various electrode configurations are shown in Fig. 5. As can be seen, for the three triangular, rectangular, and semi-circular configurations, the minimum applied voltages to separate the cells at the end of the main channel are 3.92 V, 3.15 V, and 3.52 V, respectively. Also, the buffer and sample inlet flow rates are set to 50 μL/h. This flow rate provided sufficient drag force on the cells to maintain the same moving along the microchannel and exposure time to DEP force, enough to cause separation. The simulation results for the cell's position along the y-direction show that the MDA-MB-231 cancer cells are gravitated to areas with higher electric field gradients (toward the channel wall) under the force of pDEP. Conversely, the WBCs by the nDEP force tend away from areas with higher electric field gradients (attracted to the channel center). Also simulations performed on the velocity profile showed that at the flow rate of 50 μL/h, which is the laminar flow, the projecting portions of the electrodes do not cause turbulence flow. In order to characterize the separation performance of the proposed system, the recovery rate is calculated by counting separated CTCs in the outlets and spiked CTCs in the inlet. One of the main challenges in the manipulation of CTCs is their low concentration (only a few cells per milliliter) against the high background of blood cells (approximately 5 × 10^9^ RBCs per mL and 5 × 10^6^ WBCs per mL)^4^. Therefore, it is necessary to consider these real requirements in simulation. During the simulation of this section, about 100 subtypes of WBCs with different diameters per second (Which is equal to 3.6 × 10^5^ cells per hour) and also 5 CTCs per second are entered into the sample inlet. This number of processed cells per hour, is approximately equal to the number of cells in 72 µL of a real blood sample, which is mostly WBCs and very few CTCs. It should be noted that the initial position of the cells at the inlet was randomly selected. The cells enter the microchannel within 20 s and are extracted from the outlets. The recovery rate of CTCs is obtained by counting the number of extracted CTCs in a period of 20 s at the top and bottom outlets divided by the total number of CTCs entered into the sample input during this period. Numerous simulations show the proposed system's appropriate performance, and the recovery rate for the MDA-MB-231 cells in the CTCs outlets is calculated as 95%. The simulations were repeated several times to ensure the accuracy of the recovery rate calculations, and the average of these values was reported as the final value for the recovery rate.

It is worth emphasizing that in addition to recovery rate, separation efficiency and selectivity (or purity) are used to evaluate the performance of CTC separation systems. The separation efficiency is calculated the ratio of separated CTCs in the target outlets to total number of separated CTCs in the outlets. While purity determines that how much unwanted cells are captured in the CTC outlet and is calculated as number of CTCs over total number of cells in target outlet. According to the modeling performed for the cells trajectory in the proposed systems (with the mentioned conditions in the calculation of the recovery rate) the average separation efficiency and selectivity were close to 95% and 100%, respectively.

After adjusting the voltage of electrodes to separate CTC cells with a high recovery rate, the viability of the isolated cells is discussed. As mentioned earlier, alternating electric field (AC) is used to minimize electrolysis reactions of microelectrodes and Joule heating problems. The DEP method uses high electric fields to separate cells, so cell electroporation inside the microchannel should be investigated. The magnitude of the transmembrane voltage applied to the cell near the electrodes must be such that it does not exceed a critical value. According to the trajectory of the cells shown in Fig. 5, only the MDA-MB-231 cancer cells are exposed to high electric fields, so for more detail, it is necessary to study the transmembrane voltage of the cells at different frequencies.

**Figure 6:**
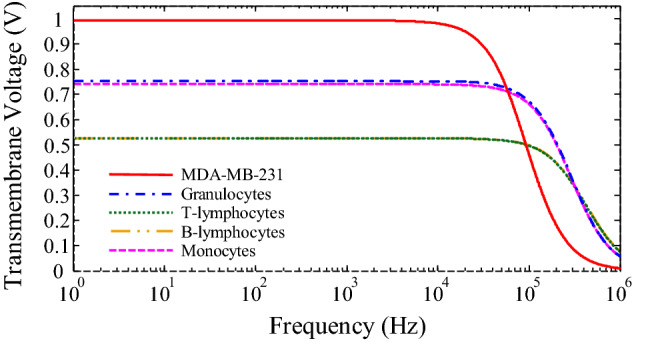
Transmembrane voltage for MDA-MB-231 and different subtypes of WBCs as a function of frequency. Parameter values used in the calculations are given in Table 1.

**Figure 7:**
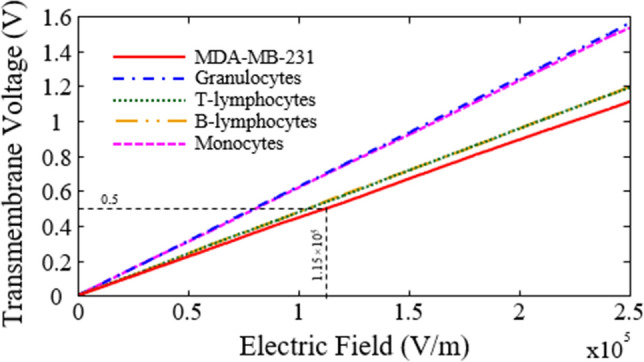
Transmembrane voltage for MDA-MB-231 and different subtypes of WBCs as a function of the electric field.

The critical transmembrane voltage of cancer cells for DC electric fields that can permeabilize the cell membrane is reported to be above 1 V^40^. According to Eq. (5) and the data in Table 1, the transmembrane voltage for MDA-MB-231 and different subtypes of WBCs as a function of frequency are plotted in Fig. 6. As can be seen for 125 kHz (separation frequency in DEP) the transmembrane voltage value of MDA-MB-231 cells has reached half of the DC value. Therefore, the transmembrane voltage induced to these cancer cells must be less than 0.5 V to maintain cell viability. Transmembrane voltage must be investigated for the various electric fields (at a frequency of 125 kHz) to obtain the maximum acceptable electric field for cell viability. Figure 7 shows the transmembrane voltage for MDA-MB-231 and different subtypes of WBCs as a function of the electric field. As can be seen, for transmembrane voltage of 0.5 V for the MDA-MB-231 cells, the electric field strength is equal to 1.15 × 10^5^ V/m. So, if the field strength lower than 1.15 × 10^5^ V/m is applied to the cells located in the microchannel, the viability of the cells will be preserved.

**Figure 8:**
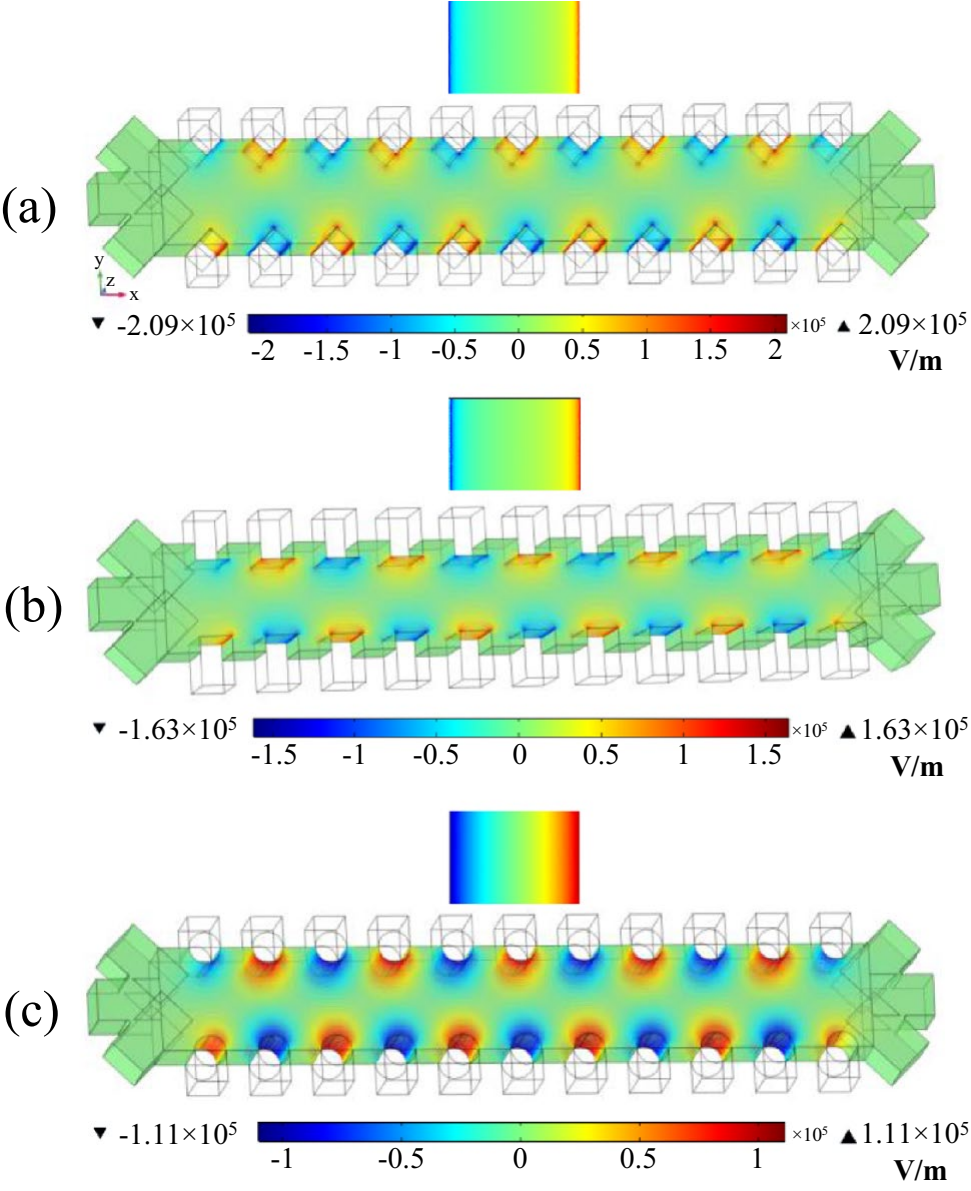
Distribution of the electric field for various electrode configurations (**a**) triangular (**b**) rectangular (**c**) semi-circular.

**Figure 9:**
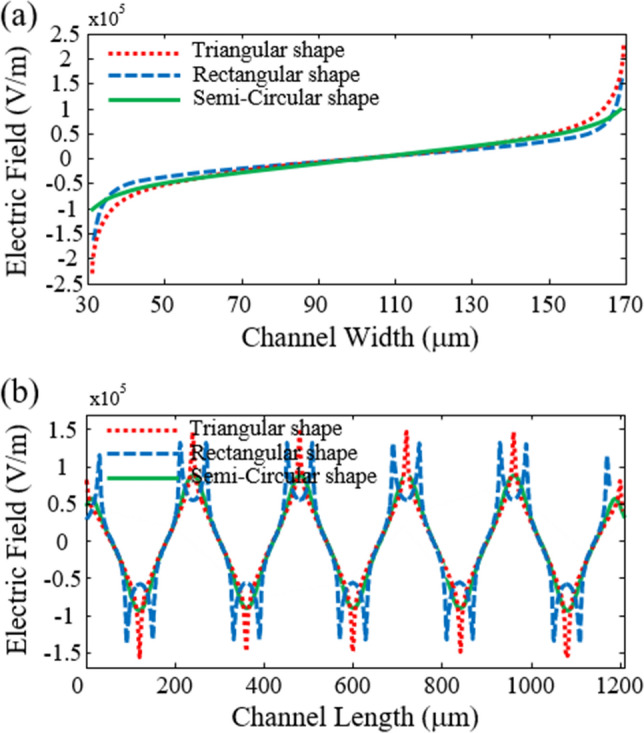
The electric field corresponds to triangular, rectangular, and semi-circular configurations (**a**) in the y-direction (**b**) in the x-direction.

The next step to minimize cells damage and attain the best electrode shapes is to survey the electric field distribution in the proposed systems. Figure 8 shows the electric field distributions for triangular, rectangular, and semi-circular configurations with separation voltages of 3.92 V, 3.15 V, and 3.52 V, respectively. As can be seen, the maximum electric field of the triangular, rectangular, and semi-circular electrodes are 2.09 × 10^5^ V/m, 1.63 × 10^5^ V/m, and 1.11 × 10^5^ V/m, respectively. Due to the presence of sharp edges in triangular and rectangular configurations, the electric field in these regions is high. Figure 9 is plotted for a better view of the electric field inside the microchannel along the y-direction (channel width) and x-direction (channel length). In the previous step, to maintain the survival of cancer cells, the electric field threshold was obtained at 1.15 × 10^5^ V/m. Figure 9a shows that the electric field near the electrodes for triangular and rectangular configurations is higher than the threshold value, and if the cells move towards the channel’s wall, a high transmembrane voltage is induced to these cells, and they are electroporated. Also, the electric field at a distance of 5 µm from the electrode is plotted in Fig. 9b, which shows that the cells are exposed to high electric fields along the channel length. The results show that the semi-circular configuration is suitable for separating MDA-MB-231 cancer cells because the electric field intensity is less than the threshold value.

It should be noted that the applied electric field for cell separation in DEP-based systems depends on various parameters, such as the number of electrodes, microchannel length, and flow rate. Although in cell separation based on triangular and rectangular configurations, by increasing the channel length and the number of electrodes as well as decreasing the flow rate, an electric field lower than the threshold value for cell electroporation can be achieved, but as the results showed, in the same operational and geometric parameters of the semi-circular configuration, an electric field above the threshold value is required to separate the MDA-MB-231 cancer cell in triangular and rectangular configurations.

**Figure 10:**
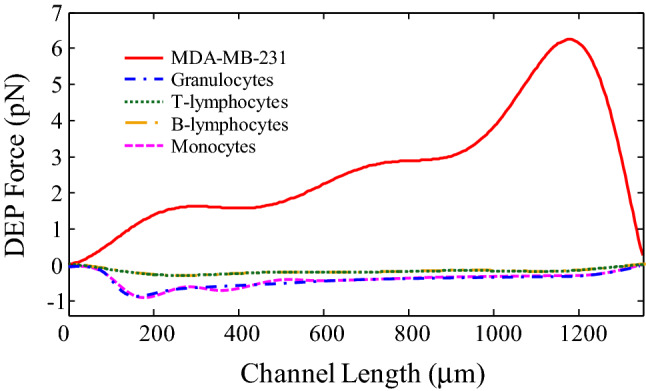
Applied DEP force on MDA-MB-231 and different subtypes of WBCs along the x-direction in a semi-circular configuration.

After selecting the semi-circular configuration for the sidewall electrodes in the proposed system, at this stage, the cells separation process by the DEP force is evaluated. Figure 10 shows the applied DEP force on MDA-MB-231 and different subtypes of WBCs along the x-direction in a semi-circular configuration. As can be seen, due to the nDEP force applied to different types of WBCs, they are directed to the center of the channel and experience the most negligible force along the channel length. Between WBCs, a more DEP force is applied to Granulocytes and Monocytes due to the larger diameter. Under the pDEP force, MDA-MB-231 cells move towards the channel walls as they approach the channel outlet experience a large DEP force due to their proximity to the electrodes. At the end of the microchannel, a DEP force of 6.3 pN is applied to these cells.

In the design of AC-DEP, operational and geometric parameters such as buffer conductivity, electrode/gap length, microchannel width, operating frequency, applied electrical voltage, and flow rate should be considered to achieve the desired level of performance criteria. Applied electric voltage and electrode/gap length have a positive effect on the performance metrics while the flow rate and microchannel width have a negative effect on the performance metrics^41^. An increase in length of electrode/spacing and applied electric voltage increases the DEP forces experienced by both CTCs and regular blood cells which leads to the splitting of the heterogeneous mixture of CTCs and regular blood cells to the appropriate streamlines. As the flow rate increases, the residence time of the cells inside the microchannel decreases and they do not reach the required streamlines, because the cells are not affected by the appropriate DEP forces. It is noteworthy that an increase in microchannel width decreases the velocity of the medium as well as the lateral distance that is needed to be transversed by cells to reach the appropriate streamline. Table 2 provides a comparison of the proposed DEP device with previously reported papers. According to the points mentioned above, each of the reported papers used different operational and geometric parameters to separate the CTCs and the applied voltage to the electrodes depends on these parameters.

**Table 2:** A comparison of proposed DEP device with previously reported papers.

Target cell line	Buffer conductivity (mS/m)	Electrode/gap length	microchannel width	Operating frequency	Applied voltage (V)	Flow rate	Separation efficiency /recovery rate (%)	References
MDA-MB-435	10	50 μm/50 μm	50 mm	20–50 kHz	4 (V_pp_)	0.2 mL/min	> 96/NA	^42^
MDA-MB-231	15	39 μm/36 μm	50 μm	40 kHz	15 (V_pp_)	6 µL/h	100 /NA	^43^
hMSC	20	50 μm/50 μm	2 mm	3 MHz	7.2 (V_pp_)	1.8 µL/min	92/NA	^44^
MDA-MB-231	15	40 μm/30 μm	2 × 65 μm	40 kHz	15 (V_pp_)	10 µL/h	–	^45^
RPMI-8402	30	50 μm/27 μm	280 μm	110 kHz	16.5 (V_pp_)	0.8 mL/h	NA/94.6	^46^
MDA-MB-435, MDA-MB-468, MDA-MB-231	30	50 μm/50 μm	25 mm	15–60 kHz	10 (V_pp_)	1.5–12 mL/min	90/NA	^47^
HOP-62 , 92 NCI-H226 , 23 EKVX	55	~ 140 μm/0 μm	200 mm	100 kHz	1.6–2.2 (V_p_)	–	99/NA	^25^
MDA-MB-231, HeLa	1	50 μm/75 μm	–	100 kHz	7 (V_pp_)	0.1 µL/min	98/NA	^48^
Yeast cell	38	100 μm/100 μm	200 μm	10 kHz	10 (V_p_)	0.15 µL/min	97/NA	^49^
MDA-MB-231	55	–	–	1 kHz	10 (V_pp_)	1.5 µL/min	90/NA	^50^
MDA-MB-231	55	60 μm/60 μm	200 μm	125 kHz	3.52 (V_p_)	50 µL/h	95/95	This study

**Figure 11:**
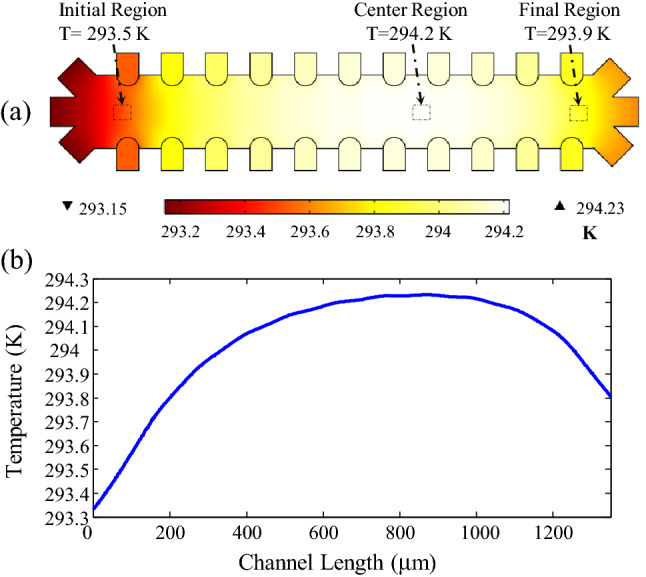
(**a**) Microchannel temperature distribution in semi-circular configuration under the voltage amplitude of 3.52 V and flow rate of 50 μL/h. (**b**) Temperature profile on the centerline.

Another major concern for maintaining cell viability in lab-on-a-chip devices based on the DEP method is Joule heating. Typically, the local electric field around the microelectrodes is relatively high. This rather sizeable electric field causes an electric current to pass through the conductive medium, resulting in significant heating of the medium and cells and denaturation of their proteins. Joule heating can be controlled and limited by optimizing the applied voltage, maintaining the medium's relatively low electrical conductivity, and configuring the electrodes. This paper uses 3D sidewall electrodes embedded in the DEP device to separate the cells with high throughput and decrease the Joule heating effect^51^. In addition, the buffer with low electric conductivity (below 100 mS/m) has been carefully selected to maintain the required physiological conditions of cell viability with and without applied voltage^52^. Figure 11a depicts the temperature distribution in all three microchannel regions of the suggested system with a voltage amplitude of 3.52 V and a flow rate of 50 μL/h. Additionally, Fig. 11b depicts the temperature profile along the centerline. As can be seen, the fluid temperature gradient within the microchannel is approximately equal to 1 K, within the safe temperature range for the cells' solution to maintain viability. Notably, temperature gradients greater than 20 K are associated with irreversible cell damage^53,54^. The supplementary materials contain details on the Joule heating analysis.

The main electrokinetic forces achieved with AC electric potentials are AC electro-osmosis (ACEO) flow, AC electrothermal (ACET) flow and dielectrophoresis (DEP). ACEO flow arises from the movement of ions in the electric double layer at the electrode/electrolyte interface, producing micro-flows because of fluid viscosity. ACEO flow generally dominates at lower frequencies because most of the applied voltage drops across the double layer, but becomes negligible above the characteristic frequency for double layer charging, $f = \left( {\sigma /2\pi \varepsilon } \right)\left( {L_{d} /l} \right)$^55,56^. For electrode spacing of 60 µm ($l$), a suspension medium with conductivity of 55 mS/m ($\sigma$), relative permittivity of 80 ($\varepsilon$), and 0.7 nm Debye length (*L*_*d*_), the double layer charging frequency is about 11.5 kHz. Therefore, for our typical operating DEP frequency (125 kHz), ACEO flow is negligible. Therefore, ACEO current is not expected to significantly affect cell mobility in this work. ACET flow is highly dependent on the fluid bulk conductivity, as power dissipation in the fluid increases with conductivity. The results of Joule heating modeling showed that the temperature rise was found to be no more than 1 K in the microchannel, displaying a minimal effect of Joule heating on the fluid flow and thus cell trajectories. The simulation results also showed that no rotational fluid flow was observed near the electrodes, thus showing the insignificant Joule heating effect on the proposed structure. The main reasons for such a minimal Joule heating effect were the use of the solution with low conductivity and the use of a glass substrate which has a good thermal conductivity in dissipating the heat from the device.

## Concluding remarks

In summary, a DEP-based microfluidic device was proposed to separate MDA-MB-231 cancer cells from different subtypes of WBCs. The main goal was to prevent cell damage in the separation process for post-processing analysis. The results showed that if an electric field higher than 1.15 × 10^5^ V/m at a frequency of 125 kHz was applied to the electrodes, the MDA-MB-231 cells would be electroporated. The cells are exposed to high electric fields inside the microchannel for a considerable time, in order to preserve the cell viability, the applied electric field must be below the threshold value. Therefore, three different configurations for the sidewall electrodes were studied. The results showed that under the same operational and geometric conditions, semi-circular electrodes needed an electric field lower than the threshold value to separate MDA-MB-231 cells at a flow rate of 50 μL/h. Also, this structure can separate MDA-MB-231 cells with a recovery rate of nearly 95%. Joule heating modeling in the proposed system showed that the fluid temperature gradient in the microchannel is about 1 K, which does not damage the cells.

## Supplementary Information

Supplementary Information.

## Data Availability

The authors declare that all data supporting the findings of this study are available within this article and its supplementary information files or from the corresponding author upon reasonable request.
