# Supplementary material for: A dielectrophoresis-based microfluidic system having double-sided optimized 3D electrodes for label-free cancer cell separation with preserving cell viability
Source: Sci Rep. 2022 Jul 15;12:12100. doi: 10.1038/s41598-022-16286-0 (PMC9287561; doi:10.1038/s41598-022-16286-0)
Supplement: Supplementary file 1 — Supplementary Information. [file 41598_2022_16286_MOESM1_ESM.docx]

A dielectrophoresis-based microfluidic system having double-sided optimized 3D electrodes for label-free cancer cell separation with preserving cell viability

V. Varmazyari^1^, H. Habibiyan^2*^, H. Ghafoorifard^1^, M. Ebrahimi^3^ and S. Ghafouri-Fard^4^

^1^ Electrical Engineering Department, Amirkabir University of Technology, Tehran, IRAN

^2^ Energy Engineering and Physics Department, Amirkabir University of Technology, Tehran, IRAN

^3^ Department of Stem Cells and Developmental Biology, Cell Sciences Research Center, Royan Institute for Stem Cell Biology and Technology, ACECR, Tehran, IRAN

^4^ Department of Medical Genetics, Shahid Beheshti University of Medical Sciences, Tehran, IRAN

***Corresponding author.**

**E-mail address:**  [habibiyan@aut.ac.ir](mailto:habibiyan@aut.ac.ir) (H. Habibiyan).

**Keywords:** *Microfluidics, Cell separation, Circulating Tumor Cell (CTC), Dielectrophoresis (DEP)*, *Cell viability*

# Numerical simulation methods and boundary conditions

This section reviews a summary of the modeling and simulation process.

## Flow Field Analysis

The physics of continuum fluid dynamics is described by the Navier-Stokes equation. In microfluidic applications since the fluid velocity of microchannel is well below the speed of sound, the flow is assumed incompressible. Also, the Reynolds number of microfluidic devices is well below unity, the viscosity term of the Navier–Stokes equation dominates and the inertia term can be ignored. So the equation can be simplified to the Stokes equation, which is a linear equation ^1^:

$\rho\frac{d}{dt}\mathbf{u}=\mathbf{-}\nabla p+\mu\nabla^{2}\mathbf{u}$ (S1)

$\nabla.\mathbf{u}=0$ (S2)

here, $\rho$, $u$, $p$ and $\mu$ are the density of the fluid, the velocity field, the pressure in the microchannel and the dynamic viscosity of the fluid, respectively. Stokes equation is used to calculate the velocity profile of the fluid in the microchannel. The boundary conditions associated with Eq. (S1) are: (1) The channel wall is a non-slip boundary. (2) The inlet fluid is controlled by the flow rate, (3) The fluid at the outlet is controlled by the pressure, and the pressure at the outlet is set to be atmospheric pressure, that is P = 0.

## Electric Field Analysis

Under steady-state conditions, the electric field distribution inside the microchannel is determined by the Laplace equation, which will be used later as an input to calculate electroosmotic flow and dielectrophoresis force ^2^.

$\nabla.\mathbf{J}=Q_{i}$ (S3)

$\mathbf{J}=\sigma\mathbf{E}+J_{e}$ (S4)

$\mathbf{E}=-\nabla\mathbf{V}$ (S5)

where $J$ is the current density, $Q_{i}$ is the charge density, $\sigma$ is the electrical conductivity, $J_{e}$ is an externally generated current density, $E$ is the electrical field, and $V$ is the applied potential. In this work, it is assumed that there is no current source or external current density. The corresponding boundary conditions are: (1) The wall surface, inlet, and outlet of the microchannel are insulated boundary conditions. (2) Sinusoidal AC voltages with the quadrature phase are exploited to the array of electrodes.

## Particle Tracing Analysis

To evaluate the performance of the microfluidic system as well as the recovery rate under the combined action of pressure-driven flow and electroosmotic flow, it is necessary to examine the path of cell movement within the microchannel. Cell trajectories can be determined based on the second law of Newton ^3^:

$\frac{d}{dt}\left( m_{c}\mathbf{v} \right)=\mathbf{F}_{\mathbf{t}}$ (S6)

$\mathbf{F}_{\mathbf{t}}\boldsymbol{=}\mathbf{F}_{\mathbf{Drag}}\boldsymbol{+}\mathbf{F}_{\mathbf{DEP}}$ (S7)

$\mathbf{F}_{\mathbf{Drag}}\boldsymbol{=}\frac{\boldsymbol{1}}{\boldsymbol{\tau}_{\boldsymbol{c}}}m_{c}\left( \mathbf{u}_{\boldsymbol{m}}-\mathbf{v} \right)$ (S8)

$\tau_{c}=\frac{\rho_{c}d_{c}^{2}}{18\eta}$ (S9)

$\mathbf{F}_{\mathbf{DEP}}=2\pi\varepsilon_{m}r^{3}Re\left\{ F_{CM} \right\}\nabla\mathbf{E}^{\mathbf{2}}$ (S10)

where $m_{c}$, $v$, $F_{t}$, $F_{\mathrm{Drag}}$, $F_{\mathrm{DEP}}$, $\tau_{c}$, $u_{m}$ and $\rho_{c}$ are mass of the cell, velocity of cell, total force acting on cell, drag force acting on cell, dielectrophoresis force acting on cell, cell velocity response time in Stokes flow, the medium velocity and mass density of cell, respectively. Also $d_{c}$, $\eta,$ $\varepsilon_{m}$, $r$, $F_{CM}$ and $E$ are diameter of cell, dynamic viscosity, the medium permittivity, cell radius, Clausius-Mossotti (CM) factor and external electric field, respectively. The dielectrophoresis force and the darg force applied to the particles are obtained by solving the electric field and the flow field, respectively. The boundary conditions associated with Eq. (S6) include: (1) The channel wall condition is a rebound. (2) The initial velocity of the inlet fluid is based on the velocity field in the medium.

## Joule Heating Analysis

The presence of an electric field within a fluid will give rise to electric current flow if the fluid has a non-zero conductivity. This leads to Joule heating of the fluid, producing a temperature gradient within the system. In the case of electrodes for electrokinetic manipulation, the heat sources can be quite localized, leading to large thermal gradients in the vicinity of the electrodes. Joule heating modeling needs to solve three equations of Navier–Stokes (for fluid flow), Laplace (for electric field), and heat transfer (for thermal study). The heat transfer equation is an energy conservation equation that says that the change in energy is equal to the heat source minus the divergence of the diffusive heat flux ^4^:

$\rho C_{P}\left( \frac{\partial T}{\partial t}+\boldsymbol{u}.\nabla T \right)+\nabla.\left( -k\nabla T \right)=Q$ (S11)

where $\rho$ is fluid density, $C_{P}$ is the heat capacity of the fluid, $u$ is the velocity field that comes from the incompressible Navier-Stokes equation, $k$ is thermal conductivity, and $Q$ represents a source term, which is calculated as follows ^4^:

$Q=\sigma E^{2}$ (S12)

here $\sigma$ is the electrical conductivity of the medium. By solving these coupled physics, cell trajectories and Joule heating in the microchannel are investigated. The boundary conditions for Stokes equation, Laplace equation, Newton’s second law and Heat transfer equation are summarized in Table S1. In this table $\boldsymbol{v}_{\boldsymbol{c}}$ is the cell velocity when striking the wall, $d_{z}$ thickness of domain in the out-of-plane direction and $q_{0}$ inward heat flux.

Table S1. Summary of the utilized boundary conditions for modeling of proposed system

| **Physics** | Inlet | Outlet | Channel Walls | Electrodes |
| --- | --- | --- | --- | --- |
| Stokes equation | Inlet velocity:  $\mathbf{u}=\left( \mathbf{u}_{x},0,0 \right)$ | Outlet pressure:  $\mathbf{p}=0$ | No slip:  $\mathbf{u}=0$ | No slip:  $\mathbf{u}=0$ |
| Laplace equation | Electric insulation:  $\mathbf{n}.\mathbf{J}=0$ | Electric insulation:  $\mathbf{n}.\mathbf{J}=0$ | Electric insulation:  $\mathbf{n}.\mathbf{J}=0$ | Electric potential:  $\mathbf{V}=V_{AC}$ |
| Newton’s second law | Inlet velocity:  $\mathbf{v}=\mathbf{u}_{\mathbf{m}}$ | Outlet velocity:  $\mathbf{v}=\mathbf{u}_{\mathbf{m}}$ | Walls velocity  $\mathbf{v}=\boldsymbol{v}_{\boldsymbol{c}}\boldsymbol{-2}\left( \mathbf{n}\boldsymbol{.}\boldsymbol{v}_{\boldsymbol{c}} \right)\mathbf{n}$ | Velocity on electrodes  $\mathbf{v}=\boldsymbol{v}_{\boldsymbol{c}}\boldsymbol{-2}\left( \mathbf{n}\boldsymbol{.}\boldsymbol{v}_{\boldsymbol{c}} \right)\mathbf{n}$ |
| Heat transfer equation | Constant Temperature  $\mathbf{T}=\mathbf{T}_{\boldsymbol{0}}$ | Outflow  $\mathbf{-n}.\left( k\nabla T \right)=0$ | Constant Temperature  $\mathbf{T}=\mathbf{T}_{\boldsymbol{0}}$ | Heat source  $\mathbf{-n}.\left( k\nabla T \right)=d_{z}q_{0}$ |

# Fabrication Process

Figure S1 shows the steps involved in fabricating 3D sidewall electrodes in the proposed DEP device. It consists of three different steps. In the first stage, 3D sidewall electrodes are made. The second step involves making a PDMS microfluidic channel from the Su-8 mold. In the final stage, the PDMS microfluidic channel is bonded to the 3D sidewall electrodes. In the first process, a seed layer of Cr/Au is deposited on the glass substrate by DC sputtering. The Cr thin film acts as an adhesion layer to improve the adhesion of the Au thin film to the substrate. To create the seed layer for electroplating, the gold layer is patterned and wet etched. After that, Omnicoat is spin-coated, which is used to lift off the negative photoresist Su-8. Later negative photoresist Su-8 is spin-coated and patterned under UV light by MASK Aligner. The pattern sample is then kept in an electroplating solution to fabricate a vertical 3D electrode. Eventually, photoresist Su-8 is removed using a lift-off process. In the second step, the Su-8 mold of the microchannel is developed on a glass substrate by photolithography. In the second step, the Su-8 mold of the microchannel is developed on a glass substrate by photolithography. Su-8 is spin-coated on glass and patterned under UV light. Then PDMS (Dow Corning's Sylgard 184) with a 10:1 ratio of base and curing agent is mixed and poured on the mold. After that PDMS is peeled off from the mold. Finally, the peeled-off PDMS is bonded to the glass substrate containing the vertical 3D electrode by oxygen plasma.

Figure S1. Fabrication process of proposed system

# Velocity profile in different meshes

| 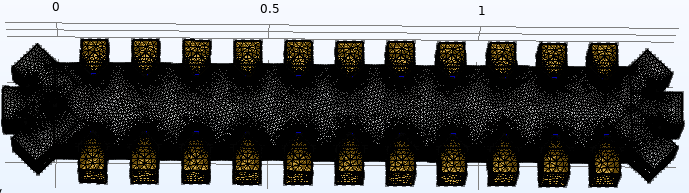  (a) | *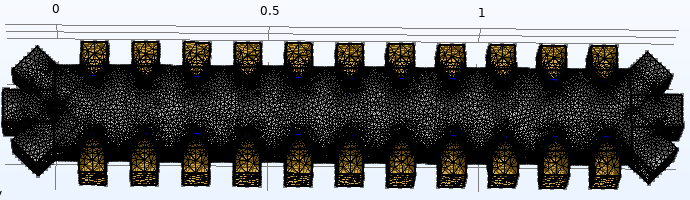*  (b) |
| --- | --- |
| *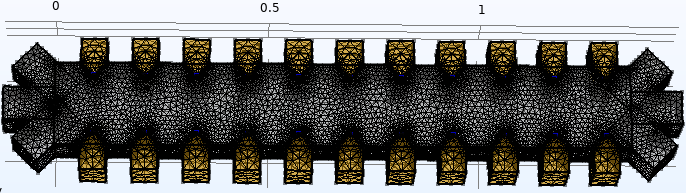*  (c) | *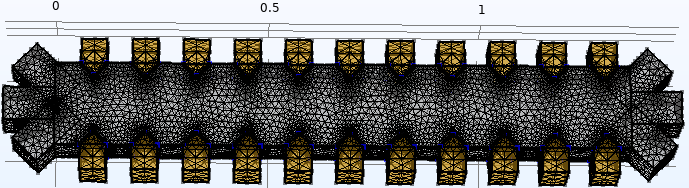*  (d) |

Figure S2 Meshing performed on the proposed structure (a) *Fine*, (b) *Normal*, (c) *Coarse* and (d) *Coarser*.

| 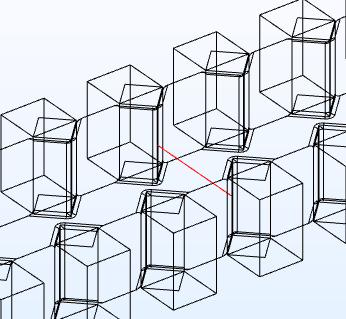  (a) |   (b) |
| --- | --- |

Figure S3 (a) Cut-line for mesh independency study (Z=100 μm and Z=810 μm) (b) Velocity profile for different meshes of *Fine*, *Normal*, *Coarse* and *Coarser*.

# References

1. Alnaimat, F., Mathew, B. & Alazzam, A. Simulation of a microfluidic device employing dielectrophoresis for liquid biopsy. *Med. Eng. Phys.* **81**, 130–135 (2020).

2. Zhang, Y. & Chen, X. Blood cells separation microfluidic chip based on dielectrophoretic force. *J. Brazilian Soc. Mech. Sci. Eng.* **42**, 1–11 (2020).

3. N, L., C, Y. & YC, L. Continuous sorting and separation of microparticles by size using AC dielectrophoresis in a PDMS microfluidic device with 3-D conducting PDMS composite electrodes. *Electrophoresis* **31**, 2622–2631 (2010).

4. Iliescu, C., Tresset, G. & Xu, G. Dielectrophoretic field-flow method for separating particle populations in a chip with asymmetric electrodes. *Biomicrofluidics* **3**, 044104 (2009).
